# Prolonged administration of maraviroc reactivates latent HIV in vivo but it does not prevent antiretroviral-free viral rebound

**DOI:** 10.1038/s41598-020-79002-w

**Published:** 2020-12-18

**Authors:** María Rosa López-Huertas, Carolina Gutiérrez, Nadia Madrid-Elena, Beatriz Hernández-Novoa, Julián Olalla-Sierra, Montserrat Plana, Rafael Delgado, Rafael Rubio, María Ángeles Muñoz-Fernández, Santiago Moreno

**Affiliations:** 1 Servicio de Enfermedades Infecciosas, Instituto Ramón Y Cajal de Investigación Sanitaria (IRYCIS) - Hospital Universitario Ramón Y Cajal, Carretera de Colmenar, Km 9.100, 28034 Madrid, Spain; 2 Unidad de Medicina Interna, Hospital Costa del Sol, 29603 Marbella, Spain; 3 AIDS Research Group, Institutd’ Investigacions Biomèdiques August Pi I Sunyer (IDIBAPS) - Hospital Clinic, Barcelona, Spain; 4 Departamento de Microbiología, Instituto de Investigación Hospital 12 de Octubre (i+12) - Hospital Universitario 12 de Octubre, Avda. Córdoba s/n, 28041 Madrid, Spain; 5 Laboratorio Inmuno-Biology Molecular, Instituto de Investigación Gregorio Marañón (IiSGM) - Hospital General Universitario Gregorio Marañón - BioBanco VIH HGM, 28007 Madrid, Spain; 6 Facultad de Medicina y Ciencias de la Salud, Universidad de Alcalá, 28871 Alcalá de Henares, Spain; 7 Present Address: Beatriz Hernández-Novoa, Medical Affairs Manager, ViiV Healthcare, Madrid, Spain

**Keywords:** Drug discovery, HIV infections

## Abstract

Human immunodeficiency virus (HIV) remains incurable due to latent viral reservoirs established in non-activated CD4 T cells that cannot be eliminated via antiretroviral therapy. Current efforts to cure HIV are focused on identifying drugs that will induce viral gene expression in latently infected cells, commonly known as latency reversing agents (LRAs). Some drugs have been shown to reactivate latent HIV but do not cause a reduction in reservoir size. Therefore, finding new LRAs or new combinations or increasing the round of stimulations is needed to cure HIV. However, the effects of these drugs on viral rebound after prolonged treatment have not been evaluated. In a previous clinical trial, antiretroviral therapy intensification with maraviroc for 48 weeks caused an increase in residual viremia and episomal two LTR-DNA circles suggesting that maraviroc could reactivate latent HIV. We amended the initial clinical trial to explore additional virologic parameters in stored samples and to evaluate the time to viral rebound during analytical treatment interruption in three patients. Maraviroc induced an increase in cell-associated HIV RNA during the administration of the drug. However, there was a rapid rebound of viremia after antiretroviral therapy discontinuation. HIV-specific T cell response was slightly enhanced. These results show that maraviroc can reactivate latent HIV in vivo but further studies are required to efficiently reduce the reservoir size.

## Introduction

The human immunodeficiency virus type 1 (HIV) currently affects 37.9 million people worldwide according to the United Nation’s HIV program, UNAIDS^1^. Antiretroviral therapy (ART) suppresses viral replication, reduces plasma viral load, and delays clinical disease progression^2^ but it does not cure the infection. Lifetime treatment is required due to viral persistence in latent reservoirs that are inaccessible to current treatments and undetectable by the immune system^3,4^. This life-long latent reservoir is quickly established in vivo after infection and consists mainly of memory resting (r)CD4 T cells harboring the viral genome integrated into their DNA^5,6^. The reservoir size in patients on ART is estimated at 10 to 100 replication-competent latent proviruses per million rCD4 T cells^7^, and these latent proviruses are sufficient to rapidly restore the infection after an ART interruption^8^.

Efforts to eradicate the HIV reservoir include use of pharmacological agents, known as latency reversing agents (LRAs)^9,10^, in an attempt to reverse proviral latency through activation of the viral gene expression without inducing T cell activation. Although HIV induces cytopathic effects, these are not potent enough to kill infected cells and therefore, viral reactivation should occur along with the action of cytotoxic effector cells, such as CD8 T lymphocytes, in order to remove HIV-expressing cells^11^. Overall the strategy is commonly known as “shock and kill”^10^. Using a stochastic model of infection dynamics, it has been estimated that a reservoir reduction greater than 10,000-fold is required to prevent rebound^12^, but how long an LRA has to be administered to ensure safe ART discontinuation remains unclear and has to be experimentally tested for each drug. To date, major problems following LRA exposure exist because ex vivo assays have shown a great diversity in HIV reactivation responses^13,14^ and have not always correlated with the in vivo ones^15^. Carefully designed clinical trials have shown the capacity of some LRAs to reactivate latent HIV in vivo^5,11–13^. Several LRAs, such as disulfiram, panobinostat, or romidepsin induces the activation of HIV transcription in ART-patients as observed by the increase in cell-associated unspliced HIV RNA (CA-US-RNA), but the decrease of the reservoir size could not be demonstrated^14–20^. These trials have included single-dose administration of a drug or, at most, multiple doses over short periods. Accordingly, a treatment modeling shows that short-term stimulation with single or combined LRAs may remove a few percentage of the latent reservoir^21^. As it is unlikely that a single treatment would significantly reduce the reservoir size, it has been suggested that multiple rounds of treatment could have a more profound effect over time, assuming that re-stimulations cumulatively reduce the frequency of latently infected cells^22^. Furthermore, the need for including analytical treatment interruption (ATI) in order to evaluate the efficacy of any therapeutic strategy design to achieve HIV eradication has been designated due to the lack of cellular biomarkers of the reservoir^23,24^.

Maraviroc is an inhibitor of HIV entry that has been approved worldwide to treat patients infected with R5-tropic HIV^25^. In the large majority of patients, R5-tropic viruses are selected during transmission and persist during primary HIV infection when the latent reservoir is established^26^. Therefore, ART intensification with maraviroc was suggested as a potential strategy for limiting reservoir size although it did not work as predicted in either chronic or acute HIV patients^27–29^. Our group conducted an open-label phase II clinical trial in order to evaluate the effects of intensification of suppressive ART with maraviroc for 48 weeks on the cellular HIV reservoir and patient follow-up was expanded for an additional 24 weeks after drug discontinuation^27,30^ (Registration: ClinicalTrials.gov NCT00795444; EudraCT 2007-003995-21). In that study, we showed that maraviroc intensification was associated with a trend to decrease the HIV reservoir size in memory CD4 T cells, to transiently increase residual viremia, and to enhance the levels of episomal two long terminal repeat (LTR) DNA circles (2-LTR-DNA), which are regarded as markers of recent infection events^27^. These effects persisted up to 24 weeks after discontinuation of maraviroc^30^ and raised the hypothesis that maraviroc could increase transcriptional activation of the latent virus and could be regarded as new LRA. Later, it was confirmed in an additional clinical trial (Trial registration: EudraCT 2012-003215-66) that maraviroc activates HIV replication in vitro^31^ and more notably, caused an increase in the expression of CA-US-RNA in rCD4T cells from HIV-infected patients on ART^32,33^. This action was mediated through the activation of the transcription factor nuclear factor (NF)-κB. Maraviroc binds to chemokine receptor (CCR)5, changes receptor conformation, and inhibits interactions with HIV, and therefore its entry into the host cell^34,35^. The effect of maraviroc on calcium flow was the only signaling pathway downstream of CCR5 receptor that was studied during commercial development of the drug^34^; therefore, its effects on NF-κB and subsequently on HIV replication were unexpected but are compatible with maraviroc interactions with signaling pathways.

In this work, we further evaluate the effects of maraviroc on HIV latent infection in vivo. In this regard, the above-mentioned clinical trial (ClinicalTrials.gov NCT00795444) was amended in order to study additional virologic parameters in stored samples and, more notably, time to viral rebound during an analytical treatment interruption (ATI) in three patients.

## Results

### Study design and subjects

This study included patients who had participated in a pilot open-label phase II clinical trial of intensification with maraviroc conducted from March 2008 to January 2015 (Trial registration: EudraCT2007-003995-21; Registration date: 24/09/2007). As described earlier, the open-label phase II clinical trial of intensification with maraviroc initially included intensification of suppressive ART with maraviroc (donated by Pfizer Inc.) for 48 weeks and patient follow-up for an additional 24 weeks after maraviroc discontinuation^27,30^. After that, the clinical trial was amended to include additional virologic measurements using the stored samples and to include an ATI of the ART that patients were receiving in order to determine the impact of maraviroc on the latent HIV infection in vivo and on viral rebound, respectively. ATI was based on the demonstration that maraviroc is capable of effectively disrupting HIV latency in vivo through NF-κB activation^32^. Study participation required that no replicative HIV could be detected in a quantitative viral outgrowth assay (QVOA) just before starting the ATI. Only three patients included in the initial clinical trial agreed to participate in the ATI substudy. The median time between maraviroc discontinuation and ART interruption was 2.5 years.

Before being included in the study, the three patients were undergoing ART with at least three drugs for no less than two years, had undetectable plasma HIV RNA (< 50 copies HIV RNA/mL) for at least two years, and the CD4 T lymphocyte count was above 350 cells/µL. This situation of adequate virologic and immunological control was maintained in every patient at the time of the ATI. The socio-demographic and clinical characteristics of the study patients at baseline are summarized in Table 1. Briefly, two patients were men, and one was a woman; the median age was 50 years [absolute range (AR): range: 51–35]. The median HIV infection time was 18.0 years (AR: 18–14), and the median time on suppressive ART was 9.75 years (AR: 8.50–16.83). Regarding the ART regimens before ATI, two patients were treated with a combination of efavirenz, emtricitabine, and tenofovir, and the other one was treated with darunavir/ritonavir, emtricitabine, and tenofovir (Table 1). All patients showed CD4 T cell counts > 500 cells/µL. The median CD4 nadir was 230 cells/µL (AR: 246–171). The median CD4 T and CD8 T cell counts were 746 cells/µL (AR: 552–989) and 857 cells/µL (AR: 590–883), respectively. The CD4/CD8 ratio was 0.8 (AR: 0.6–1.6). The median percentage of CD38+/HLA-DR+/CD4+ T cells and CD38+/HLA-DR+/CD8+ T cells was 2.3 (AR: 1.7–2.9) and 2.9 µL (AR: 2.1–5.6), respectively.

**Table 1:** Socio-demographic and clinical characteristics of the study patients at the time of starting the analytical treatment interruption.

	Patient 1	Patient 2	Patient 3
Gender	Female	Male	Male
Age (years)	51	50	35
Time of HIV infection (years)	18	18	14
Time on ART (years)	9.75	16.83	8.50
Current ART regimen	EFV + FTC + TDF	DRVr + FTC + TDF	EFV + FTC + TDF
Nadir CD4 (cells/µL)	171	246	230
CD4^+^ T cell counts (cells/µL)	746	989	552
CD8^+^ T cell counts (cells/µL)	857	590	883
CD4:CD8 Ratio	0.8	1.6	0.6
CD38^+^/HLA-DR^+^/CD4^+^ T cells (%)	1.7	2.3	2.9
CD38^+^/HLA-DR^+^/CD8^+^ T cells (%)	2.1	2.9	5.6
IUPM	< 0.012	< 0.012	< 0.012
Episomal2 LTR DNA circles	Undetectable	Undetectable	Undetectable
HIV tropism	R5	R5	R5

*ART* antiretroviral treatment, *DRVr* ritonavir-boosted darunavir, *EFV* efavirenz, *FTC* emtricitabine, *TDF* tenofovir, *IUPM* infections units per million cells.

The number of memory CD4 T lymphocytes that were latently infected with replication-competent HIV was measured at baseline using a QVOA as previously described^3,27,36,37^. Results confirmed that the reservoir size persisted below the limits of detection, which was 0.012 infections units per million cells (IUPM) in the three patients (Table 1). At baseline, no patient showed the 2-LTR-DNA. All of the patients had wild-type CCR5 genotypes (Table 1), which was a requisite to be included in the initial study.

### Cell-associated unspliced HIV RNA is enhanced during maraviroc intensification but reduced after treatment discontinuation

**Figure 1:**
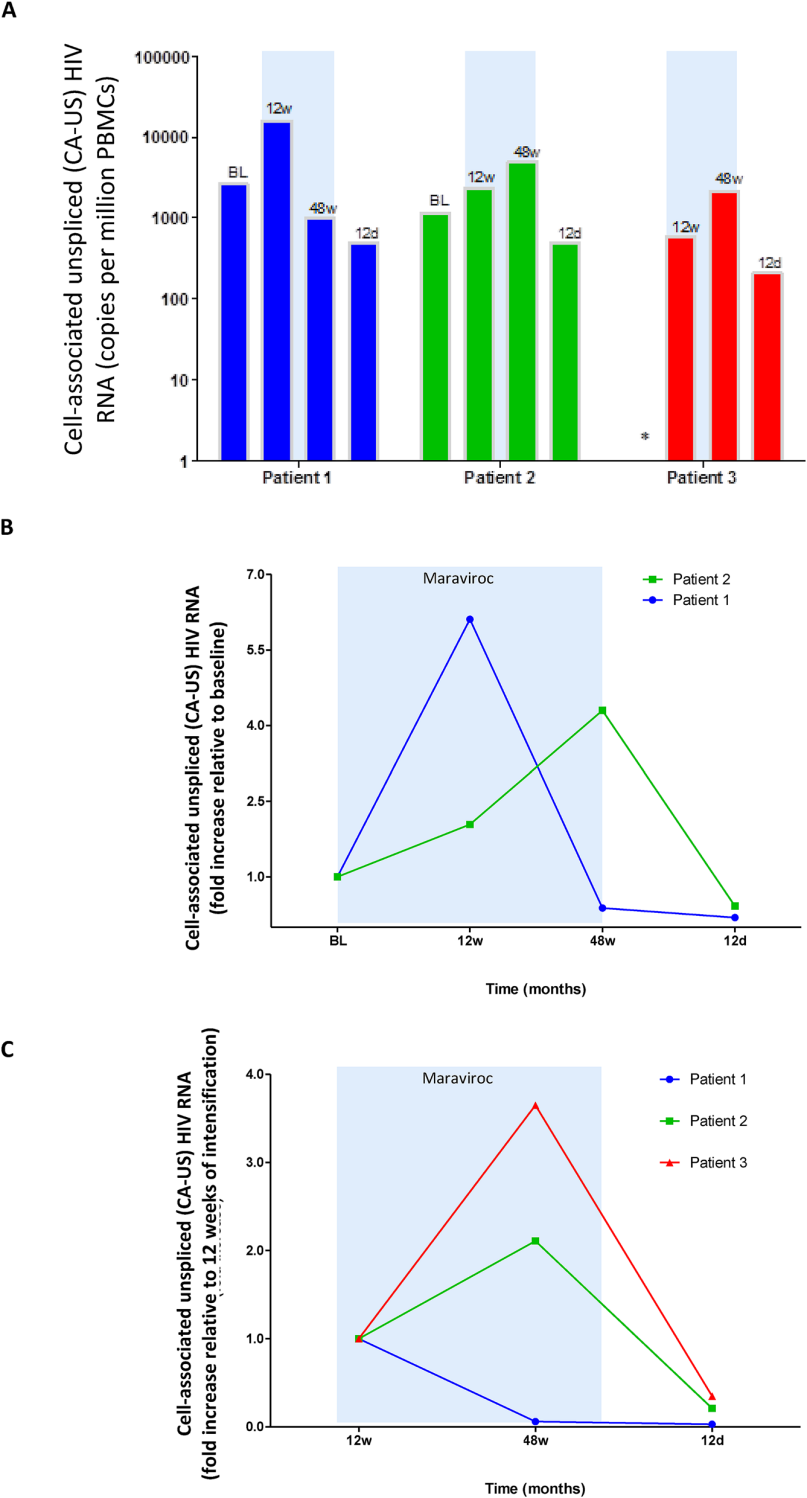
Levels of cell associated unspliced HIV RNA in PBMCs isolated from HIV-infected patients on ART who were under intensification with maraviroc. (**A**) Copies per million peripheral blood mononuclear cells (PBMCs), (**B**) relative fold change with respect to baseline and (**C**) relative fold change with respect to 12 weeks of treatment intensification. The blue shaded box shows the time on maraviroc intensification. BL, baseline; 12w, 12 weeks on maraviroc; 48w, 48 weeks on maraviroc; 12d, 12 weeks after discontinuation of maraviroc. *Baseline sample in patient 3 was not available.

CA-US-RNA has been appointed as an indicator of viral transcription and ongoing viral production from latently infectedcells^38^. CA-US-RNA was measured in human peripheral blood mononuclear cells (PBMCs) isolated before intensification with mararivoc, after intensification for 12 and 48 weeks, and again at 12 weeks after maraviroc discontinuation. Based on this schedule, CA-US HIV RNA was measured before starting ATI. Unfortunately, the baseline of patient-3 could not be measured due to sample limitations. The expression of CA-US-RNA, expressed as copies per million PBMCs, showed a slight increase during maraviroc intensification for 12 weeks in patient-1 and patient-2 and was further increased throughout the follow-up in patient-2 and patient-3 (Fig. 1A). Relative data, shown as fold increase relative to baseline, are summarized for patient-1 and patient-2 (Fig. 1B); the fold increase relative to 12 weeks of intensification is also shown for the three patients (Fig. 1C). Briefly, the CA-US-RNA expression was enhanced 6.11-fold after 12 weeks in patient-1, but it reached levels lower than those shown at baseline at 48 weeks of maraviroc intensification. Although CA-US-RNA levels increased only 2.04-fold compared to the baseline in patient-2, it was further enhanced up to 4.30-fold after 48 weeks of drug treatment. In patient-3, CA-US-RNA expression levels at 48 weeks were 3.65-fold higher than at 12 weeks of maraviroc exposure. Considerably, there was a decrease in CA-US-RNA after maraviroc discontinuation in patient-1 and patient-2, as levels were 0.19- and 0.42-fold reduced, respectively, in comparison to baseline levels. In patient-3, levels were 0.35-fold reduced in comparison to levels at 12 weeks of intensification. Similarly, CA-US-RNA expression was reduced 0.03- and 0.21-fold in patient-1 and patient-2, respectively, when compared versus 12 weeks of drug exposure. These results confirmed previous data indicating that administration of maraviroc can increase HIV transcription from latency in HIV patients and therefore may potentially decrease the latent reservoir in vivo*.*

### HIV rapidly rebounded in the maraviroc-treated patients included in the analytical treatment interruption

**Figure 2:**
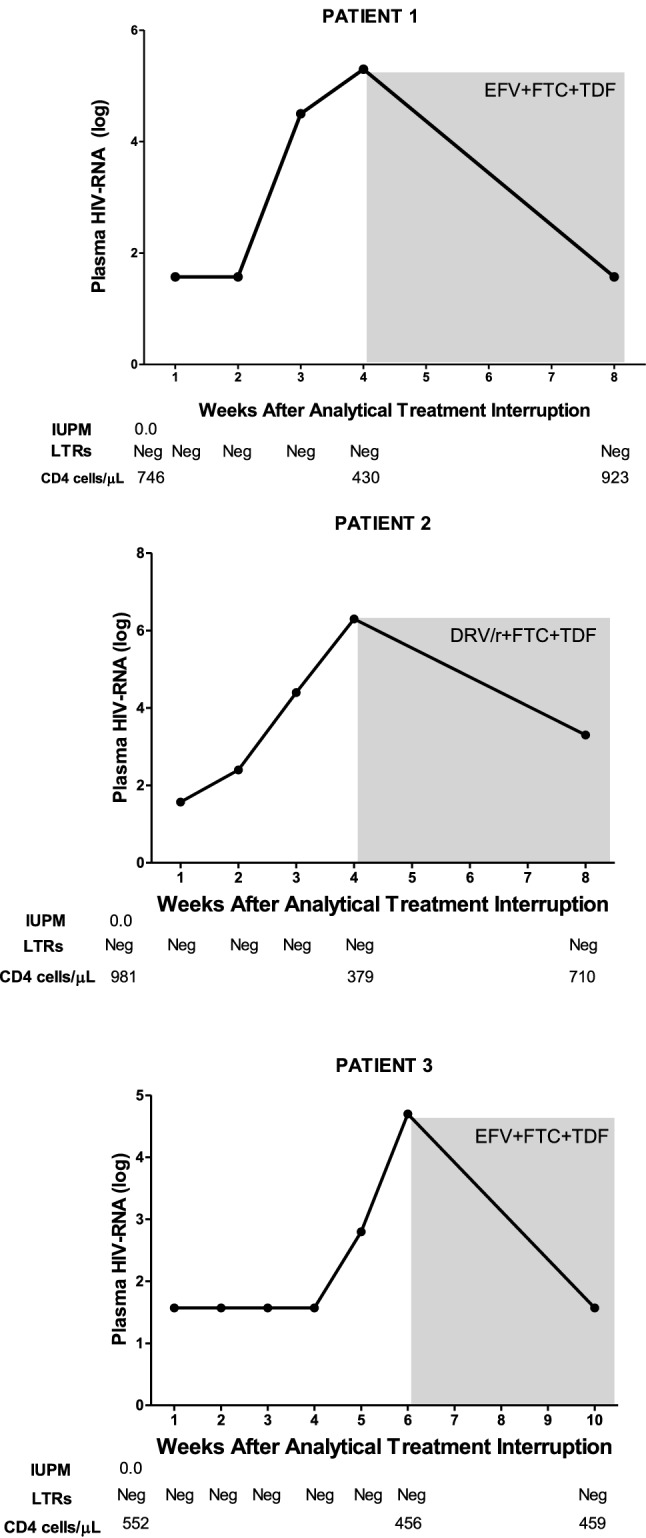
Virologic evolution of HIV rebounded in the maraviroc-treated patients who underwent into the analytical treatment interruption shown in terms of conventional viral load (log plasma HIV RNA), infections units per million cells (IUPM), episomal two LTR-DNA circles (2LTRs), and total CD4 T cell count (CD4 cells/µL). Measurements for each parameter were done at the indicated time. The grey shaded box shows the time on ART restart. EFV, efavirenz; FTC, emtricitabine; TDF, tenofovir; DRV/r, ritonavir-boosted darunavir.

The three patients underwent ATI once that it was confirmed that maraviroc could disrupt HIV latency and that replicative-competent HIV was undetectable. Patients were closely monitored during the ATI, including a plasma HIV RNA test once a week to detect viral rebound as soon as possible. In the case of HIV rebound, it was scheduled to restart the previous ART immediately after confirming a viral load determination higher than 200 copies/mL (2.30 log10). The detection limit of the assay was 1.57log10 HIVRNA copies/mL. Viremia rebound was rapidly detected in all of the patients, at weeks 3, 2, and 5 after ART interruption for patient-1, patient-2 and patient-3, respectively (Fig. 2). Therefore, the median time of HIV rebound was 3 weeks (21 days). The highest values of viremia before re-initiation of the previous ART were 5.8, 6.3, and 4.7 log10 HIV RNA copies/mL at 4, 4, and 6 weeks in patient-1, patient-2 and patient-3, respectively (Fig. 2). CD4 T cell counts just before starting ATI before were 746, 981 and 552, respectively for patient-1, patient-2 and patient-3 (Fig. 2). CD4 T cell counts at the end of the study were 923, 710 and 459, respectively for patient-1, patient-2 and patient-3. Plasma levels of antiviral drugs were undetectable one week after drug discontinuation in all cases. After the confirmation of viral rebound, the three patients resumed previous ART with good response. No patient developed an acute retroviral syndrome. Genotyping of the rebounded HIV showed no resistance mutations and therefore starting the previous ART led to the subsequent control of plasma viremia within the following 4 weeks in all of the patients.

### T cell specific responses against some peptide pools of HIV was weak but increased at the end of the study in two out of three patients

**Figure 3:**
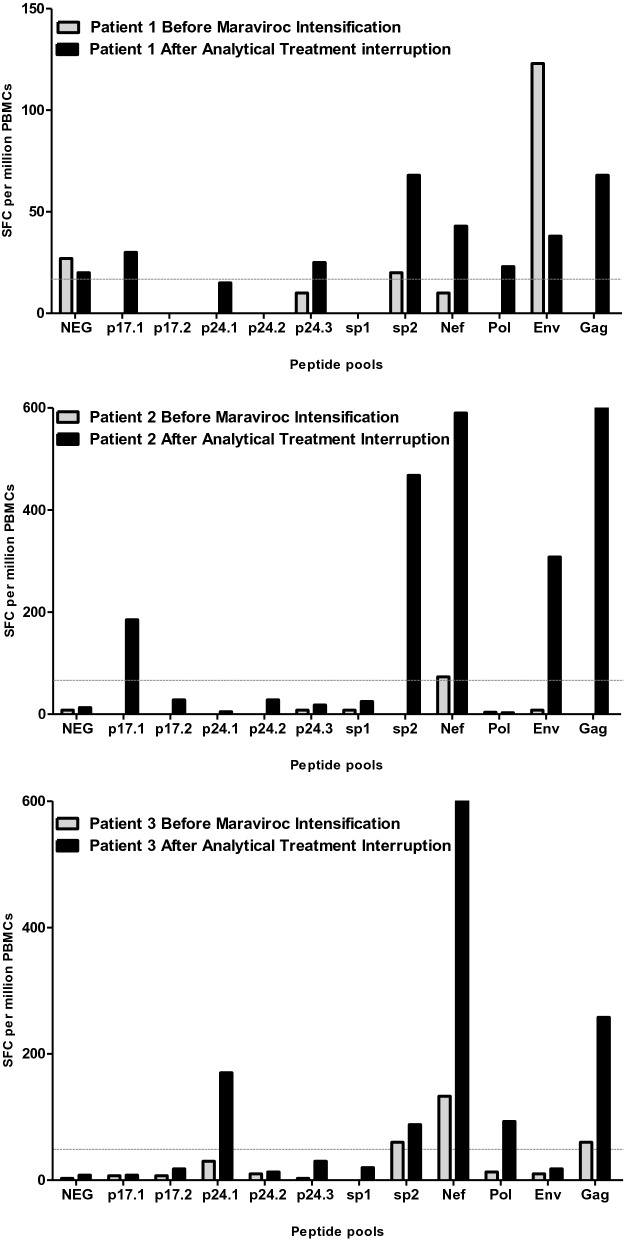
HIV specific T lymphocyte response. The ELISpot assay, that quantifies interferon-γ secretion against different peptide pools covering *p17, p24, sp1, sp2, gag, pol, nef* and *env* sequences, was used. Samples were taken before the intensification with maraviroc during the initial study (grey bars) and when antiretroviral treatment was resumed after the ATI during the amended study (black bars). Data are are shown as the number of cells secreting interferon-γ against the indicated peptide per 10^6^ peripheral blood mononuclear cells (PBMCs). The dotted grey line indicates the threshold for positivity (50 SFC per million PBMCs). SFC, spot forming cells.

After reversal of HIV latency, the cytopathic effects of the virus were not potent enough to kill infected cells^11^ and therefore the removal of HIV-expressing cells must have occurred via the action of an immune cytotoxic effector, such as CD8 T lymphocytes^11,39^. Therefore, the specific responses of CD8T lymphocytes against HIV was studied in these patients using an ELISpot assay that quantifies interferon-γ secretion against different peptide pools covering *p17, p24, sp1, sp2, gag, pol, nef*, and *env* sequences. The methodology used involved at least 85% of CD8 T cell responses^40,41^. However, all studies were conducted with unfractionated PBMCs and, therefore, responses are described as HIV-specific T cells. Measurements were performed at two time points: (1) before the intensification with maraviroc during the initial study and (2) the point at which ART was resumed after the ATI during the amended study. The three patients had very weak HIV-specific T cell responses before intensification with maraviroc (Fig. 3). Although low, these responses had increased at the end of the study in some peptide pools, such as *sp2*, *nef* and *gag*, mainly in patient-2 and patient-3 but not in patient-1 (Fig. 3). Briefly, specific responses against *p17.1*, *sp*2, *nef*, *env*, and *gag* were enhanced 23.13-, 58.50-, 73.75-, 38.50-, and 81.30-fold, respectively, after maraviroc exposure and ATI intervention in patient-2. Similarly, specific responses against *p24.1*, *sp*2, *nef*, *pol*, and *gag* were enhanced 56.67-, 29.33-, 214.33-, 31.00- and 86.00-fold, respectively, in patient-3.

### HIV clones found in PBMCs were not present in plasma at the time of viral rebound

**Figure 4:**
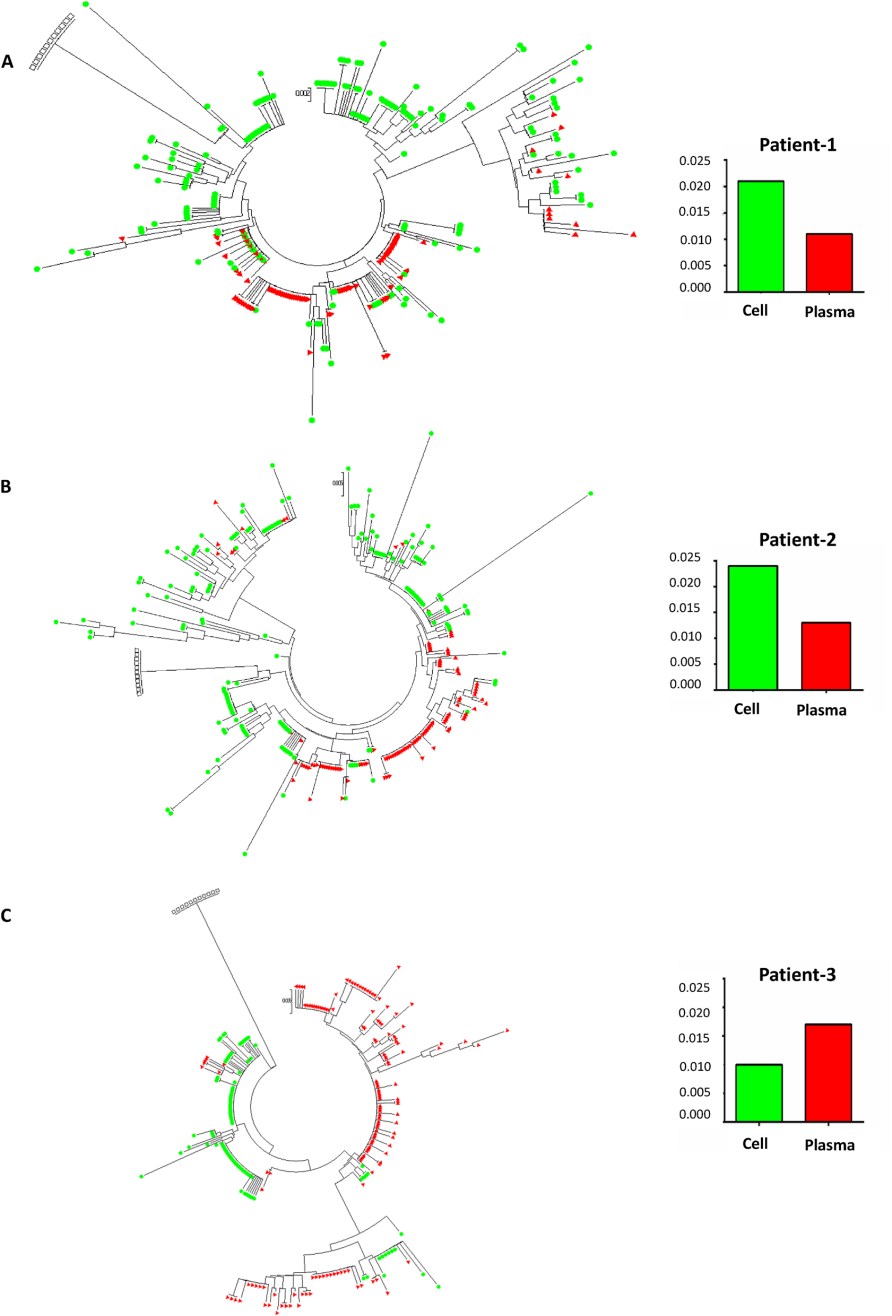
Phylogenetic analysis of the latent pro-viruses contributing to HIV rebound after analytical treatment interruption. (**A**) Patient 1; (**B**) Patient 2; (**C**) Patient 3. HIV protease sequences were obtained from DNA isolated from peripheral blood mononuclear cells obtained while patients were on antiretroviral treatment and maraviroc intensification during the initial study (green) and from plasma RNA after viral rebound at analytical treatment interruption during the amended study (red). Open squares represent the HIV reference sequence HXB2. Histograms represent the average pairwise distance (APD) between HIV sequences on cellular and plasma samples.

The diversity of the latent pro-viruses contributing to HIV rebound after ATI was evaluated through a phylogenetic analysis based on deep sequencing of the protease region. The analysis was done on cell-associated HIV DNA from frozen PBMCs obtained from patients while they were on ART during maraviroc intensification as well as on plasma HIVRNA obtained at two time points: (1) just after HIV rebound and (2) a week later when viral rebound was confirmed. Lower diversity was observed (average pairwise distance [APD]) in HIV genomes sequenced from plasma than from cells in patient-1 and patient-2 but not in patient-3 (Fig. 4). Many HIV clones found in PBMCs were not present in plasma at the time of viral rebound; this observation was especially significant for patient-1 and patient-2.

## Discussion

HIV infection remains currently incurable due to viral persistence in latent reservoirs and therefore, patients require lifelong antiretroviral treatment. Clinical trials are the only experimental approach for investigating whether drugs aimed at disrupting HIV latency in vivo are effective or not^18^. The results shown here confirm that therapeutic doses of maraviroc can disrupt the quiescence of latent HIV proviruses within rCD4 T cells, although the sample size of our study was quite limited. It quite unlikely that results are stochastic because the increase of HIV transcription after maraviroc administration in patients has already been described in previous clinical trials conducted by our group and others^32,33^. Traditionally, the HIV reservoir mainly consists of several populations of memory rCD4 T cells, such as stem central, transitional memory and effector memory cells^5,6^. However, recent studies have suggested that viral latency is not restricted to non-activated and quiescent CD4 T cells and may also be established in activated and proliferating cells^42^, suggesting that the number and type of CD4 T cells capable of supporting viral latency is larger than initially assumed. Unfortunately, this hypothesis arose after our study and could not be tested in our patients.

Multiple rounds of treatment with LRAs have been suggesting as a strategy to cumulatively reduce the reservoir size^22^. However, most studies have included only single-dose administration of a drug or, at most, multiple doses over short periods^16–19^. Prolonged administration of maraviroc for 48 weeks increased HIV transcription as measured via CA-US HIV-1 RNA levels, confirming data previously observed by our group and others^32,33,43^. This increase was within the range of that observed in studies with some histone deacetylase inhibitors (HDACi), such as vorinostat or panobinostat^16,18,19^. Moreover, maraviroc-mediated HIV reactivation persisted during the administration of the drug, increasing in two out of three patients until week 48 of administration and then decreased when the drug was discontinued. These findings may be important for the design and evaluation of maraviroc as an LRA in clinical trials as each drug may display a different pattern. For example, reactivated HIV persisted after 8 weeks of panobinostat treatment although intermittent administration of the drug was scheduled in this clinical trial^18^. However, the decrease in the reservoir size could not be demonstrated, similar to what has been shown with most tested LRAs^17–20,44,45^. Consequently, at present, it is assumed that a combination of LRAs that exploit different cellular pathways is required to induce the complete expression of replication-competent proviral genomes and therefore, to effectively reduce the size of the HIV reservoir *in vivo*^13,46,47^. The well-known tolerability and safety of maraviroc make it a good candidate for safe and active combinations of LRAs aimed at the cure of HIV infection.

Furthermore, the so-called “shock and kill” strategy^10^ aimed at curing HIV includes the administration of any LRA in combination with an additional action that enhances CD8 T lymphocytes so that HIV-expressing cells are removed. It is believed that CD8 T cell exhaustion prevents the control of the infection in HIV-infected patients^48–51^. Therefore, it is essential to describe the effects of LRAs on CD8 T lymphocyte functions so that their therapeutic potential can be determined^52,53^. In our study, the HIV-specific T cell responses, assumed to be mainly from CD8 T lymphocytes^40,41^, slightly increased in two out three patients after maraviroc intensification and ATI intervention. This may not directly be due to maraviroc but to active viral replication. Because the median time between maraviroc discontinuation and ART interruption was 2.5 years we cannot reject HIV-specific immunity had already been vanished. However, it is also plausible that the effects of maraviroc on CD8 T cells may persist during long periods through the persistence of long-lasting memory cells that acquire multiple unique functional features which make them able not only to respond to, but also to actively provide different signals ultimately culminating in host protection^54^. A remarkable issue is that HIV-specific T cell responses are not reduced after maraviroc intensification, in agreement with data from in vitro assays that we have recently published^55^. This confers maraviroc an important advantage over other well-known LRAs as panobinostat or disulfiram as both of them dramatically reduces in vitro the cellular viability of CD8 T cells isolated from both healthy donors^52,56^ and HIV-infected patients on ART^55^, even after a short-term stimulation. As a result, the specific antiviral function of CD8 T cells is inhibited by some histone deacetylase inhibitors, including panobinostat^57^, which is a profound limitation for the implementation of in vivo trials.

Rapid plasma viral rebound occurs upon ART interruption in the vast majority of infected individuals^58^. Plasma viremia levels remain the only clinically relevant virologic marker that is currently available and therefore, the efficacy of any therapeutic strategies designed to achieve ART-free virologic remission can only be adequately assessed with ATI with extensive monitoring of virologic and immunological parameters^23,59^. Recent studies have demonstrated that short-term ATI followed by immediate re-initiation of ART after HIV rebounded did not lead to permanent expansion of the HIV reservoir or irreversible damage to the immune system of infected individuals^60–64^ and therefore, it is justified and safe for clinical trials of potentially curative interventions to include ATI^59^. In this study, QVOA could not be performed to quantify HIV replication due to sample limitations. Therefore, including ATI in this clinical trial was required to evaluate the efficacy of maraviroc as an LRA in vivo. In order to do that, a previous open-label phase II clinical trial of ART intensification with maraviroc for 48 weeks was amended to include ATI and to use the remaining samples before ATI, as it has been done in other studies^62^.

During ATI, reactivation of a single latently infected cell can lead to HIV rebound. Control of viremia of the rebounded viruses depends on both the strength of cytotoxic T lymphocytes and the size of the HIV reservoir^65^. Furthermore, the total inducible reservoir is significantly smaller in women than in men^66^. Virologic rebound was detected in the three patients evaluated, all of the men, soon after ART cessation, and the time to rebound was similar to previous studies in cohorts of HIV infected individuals^59,61,64^ or, for instance, in one study using panobinostat as an LRA^18^. In our study, this observation may be explained from at least three different perspectives: Firstly, it is very likely that maraviroc as an LRA is not potent enough and higher doses or combinations of drugs should be used as discussed above. Secondly, there could be lack of capability of targeting the pool of latently infected cells^67^, and accordingly, maraviroc only could reactivate a minority of latent pro-viruses. Supporting this argument, we have found a low diversity of viral clones in plasma after the rebound in two out of three patients analysed. Patients included in the primary study were infected with wild-type CCR5 genotypes according to the fact that most of the founder viruses establishing the latent reservoir during early infection are CCR5-tropic^26,68^ and persisted when the amended study started. However, HIV clones found in PBMCs were not present in plasma at the time of rebound, especially in two patients, suggesting that rebounded viruses do not come from circulating blood but from lymphoid nodes or gut-associated lymphoid tissue (GALT)^69^, that are regarded as the main anatomical HIV reservoir in ART-treated patients due to low ART concentrations in these areas^70,71^. Thirdly, HIV-specific immune responses, required for the clearance of the latent reservoir^11,39^, seems not to be potent enough as ELISpot results showed only an slight increase in these responses. However, the assay used involved PBMCs instead of purified CD8 T cells due to sample limitations and therefore cytotoxic responses may come also from other cell types as, for instance, Natural Killer cells. Anyhow, combining maraviroc with a treatment-enhancing cytotoxic functions seems necessary in order to efficiently reduce the reservoir size after reactivation.

The results presented in this study show that maraviroc reverses HIV latency in vivo and confirm that it may be considered a new LRA. Although the effect persists during the administration of maraviroc, prolonged administration of the drug fails to impact the time to viremia rebound after ART interruption, which can be due to either insufficient potency of the drug, the lack of a strong HIV specific response, or both. Furthermore, time to rebound after ATI was similar to that observed in ART-treated patients who have not received any therapeutic intervention, as discussed above^59,61,64^, showing that long-term intensification with maraviroc did not significantly reduce the HIV reservoir size. Because changes in HIV transcription were also modest, it seems that the reservoir size was balanced from maraviroc intensification to ATI, probably through homeostatic proliferation. Considering the results shown is this work, ATI should take place earlier in future studies. Higher cell-associated unspliced HIV RNA (CA-US-RNA) levels correlated with faster time to rebound^72^. Therefore, ATI cannot be implemented just after maraviroc intensification when CA-US-RNA are the highest due to viral reactivation but at least 12 weeks after the intervention when CA-US-RNA were similar to levels at baseline.

The effect of maraviroc on initiating HIV transcription from latent reservoirs was similar after administrating the drug for 10 days^32,33^ or for 48 weeks and longer administration did not result in HIV reservoir disruption. Therefore, it seems that combining maraviroc with other LRA or with other experimental strategy will be more suitable than extending maraviroc administration longer in order to disrupt latency. So far, in vitro studies looking for combination of maraviroc with other LRAs has not been successful^31,55^.

A major limitation of our study is that it only included three participants. Therefore, clinical trials including a higher number of patients treated with maraviroc fare needed to confirm the results shown here. The strength of the results would have been also increased using a control group or samples from the patients included in the initial study who did not agree to participate in the amended one, but it could not be done due to ethics concerns. Further characterizing the reservoir size and the proportion that is able to induce new infections in maraviroc treated patients as well as determining the changes induced in the genomic profile and the functional effects of these changes in immune system may be also relevant but represent themselves new clinical trials that should be performed in the future.

Maraviroc is a CCR5 inhibitor approved for clinical use as an antiretroviral drug and therefore, a new use for this drug can be rapidly authorized. An additional advantage of maraviroc is that specific T cell responses are not reduced after long-term exposure contrary to other common LRAs. Additionally, further clinical trials combining maraviroc with other LRAs and/or with strategies to enhanced cytotoxic responses are warranted.

## Materials and methods

### Plasma HIV RNA measurement

The plasma viral load was quantified using the VERSANT HIV RNA kPCR system (Siemens Healthcare Diagnostics Inc., Tarrytown, NY) with an assay limit of quantization of 37 copies HIV RNA/mL, equivalent to 1.57 log10 HIV RNA copies/mL.

### Quantification of CD4 T cells harbouring replication-competent HIV

The detection of CD4 T cells harbouring latent and replication-competent HIV was determined via a QVOA as previously described^3,37^, with some minor modifications^27,36^. This assay involves an enhanced culture of highly enriched rCD4 T cells and provides a precise minimal estimation of the HIV reservoir in rCD4 T cells, which constitutes the principal viral reservoir^5,6^. The QVOAis widely accepted and considered as the gold-standard formeasuring the frequency of productively- and latently-infected cells in clinical settings^73^. rCD4 T cells were isolated from total PBMCs via negative selection of CD3^+^/CD4^+^/HLA-DR^−^/CD25^−^ T cells using magnetic beads according to manufacturer’s recommendation (MiltenyeBiotec, Bergisch Gladbach, Germany). Cells were then placed in a duplicate five-fold serial dilution cultures, ranging from 25 × 10^6^ to 320 cells. Irradiated PBMCs from healthy donors were added at ten-fold to each culture with phytohaematoglutinin (PHA, 1 mg/mL) (Sigma-Aldrich, St. Louis, MO, USA) and recombinant interleukin 2 (IL-2, 100 U/mL) (Sigma-Aldrich) in order to achieve efficient cell activation. On days 15 and 21, culture supernatants were tested for HIV replication trough the presence of viral antigen using the HIV p24 antigen assay kit (Innogenetics, Barcelona, Spain). The frequency of infected cells was determined using the maximum likelihood method and was expressed as infectious units per million (IUPM) of rCD4 T cells with a limit of detection of 0.012 IUPM.

### Quantification of cell-associated unspliced HIV RNA

A semi-nested quantitative PCR was performed to quantify CA-US-RNA as previously described^74^. Briefly, total RNA was extracted from stored PBMCs using RNeasy Mini Kit Qiagen (Hilden, Germany), and the eluted cellular RNA was directly subject to two rounds of PCR amplification. The primer pair used in the first PCR consisted of MH535 (5′-AACTAGGGAACCCACTGCTTAAG-3′) and SL20 (5′-TCTCCTTCTAGCCTCCGCTAGTC-3′) and amplified a region within the HIV *gag* gene. The first PCR round was performed using these conditions: (1) 95 °C for 10 min; (2) 15 cycles of 94 °C for 20 s; (3) 55 °C for 40 s; and (4) 72 °C for 40 s. The product of the first PCR was subsequently used as a template for the second semi-nested PCR amplification, which was performed on a real-time LightCycler 480 machine (Roche Life Sciences, Penzberg, Germany) using SYBR Green detection. The primer pair was SL20 and SL19 (5′-TCTCTAGCAGTGGCGCCCGAACA-3′). Triplicates were conducted for each tested experimental condition. The second PCR settings were as follows: (1)95 °C for 10 min; (2)45 cycles of 94 °C for 20 s; and (3) 55 °C for 40 s. Synthetic runoff RNA transcripts, corresponding to the HIV *gag* region, were used as external standards. These RNA standards were kindly gifted by Professor Sharon R. Lewin (Doherty Institute, Melbourne, Australia). Serial dilutions of standards between 1 and 4, 4 × 10^11^ input copies were made. The amplicon sizes were 286 bp for the first PCR and 160 bp for the second one. HIV RNA copy numbers were standardised to cellular equivalents using RNA concentration (assuming that 1 ng RNA corresponds to 1000 cells^75^, which has been shown to correlate with levels of glyceraldehyde phosphate dehydrogenase [GAPDH] RNA^76^). PCR results are expressed in CA-US-RNA copies per million PBMCs.

### Detection of episomal two LTR DNA circles

The existence of 2-LTR-DNA was checked via nested PCR as described previously^27^. DNA was extracted from 5 million PBMCs using QIAprep Spin Miniprep following manufacturer’s protocol for low copy number plasmids in order to maximize the recovery of 2-LTR-DNA^77^. A nested PCR flanking the junction of the 2-LTR-DNAwas designed. In the first round, episomal DNA was amplified using the following pair of primers: forward, 5′-TAAGATGGGTGGCAAGTGGTCA-3′ and reverse, 5′-TCTACTTGTCCATGCATGGCTT-3′. The second PCR round used a set primers spanning the unique junction formed by ligation of 5′ and 3′ LTR sequences: forward, 5´-AATCTCTAGCAGTACTGGAAG-3′ and reverse, 5′-GCGCTTCAGCAAGCCGAGTCCT-3′. PCR products were analyzed on a 1% agarose gel stained with GelRed (Biotium, Hayward, California, USA).

### Measurement of antiretroviral drug levels in plasma

Five milliliters of total blood were collected without anticoagulant for pharmacokinetics assays before starting the ATI and weekly thereafter. Blood samples were centrifuged and plasma was stored at − 80 °C until posterior analysis. Concentrations of the antiretroviral drugs included in the treatment regimens were measured by high-performance liquid chromatography (HPLC).

### ELISpot assay

ELISpot assay was performed to measure the numbers of PBMCs producing interferon (IFN)-γ as a response against HIV sequences^78^. Briefly, experiments were performed with cryopreserved PBMCs using 10 pools of peptides, each consisting of 15-mers overlapping 11 peptides, grouped in pools of 10 to 12 peptides each, and covering the whole HIV *gag*, *nef*, *pol*, and *env-gp41* sequences. Negative control responses were obtained with non-stimulated PBMCs. Positive controls were PBMCs stimulated with PHA and a pool containing 32 human leukocyte antigen (HLA) class I peptides from cytomegalovirus (CMV), Epstein-Barr virus (EBV), and influenza virus (CEF pool obtained from the National Institutes of Health (NIH) AIDS Research and Reference Research Program). The assay was done in 96-well polyvinyl difluoride (PVDF) microtiter plates coated overnight with a mAb specific for human IFN-γ (mAb B-B1, Diaclone, BioNova Cientifica, Spain). PBMCs resuspended in RPMI medium plus 10% fetal bovine serum were plated in the presence of different peptide pools (2 μg/mL, final concentration) and incubated overnight at 37 °C, 5% CO_2_. Plates were developed using biotinylated anti-human IFN-γ streptavidin conjugated to alkaline phosphatase (Amersham Biosciences, UK) and chromogenic substrate BCIP/NBT (Sigma-Aldrich). Spot-forming cells (SFC) were counted using an AID ELISPOT reader (Autoimmun Diagnostic GmHb, Germany). Results were expressed as the number of SFC per million of PBMCs after subtracting the background. The positivity threshold for each peptide pool or antigen was defined at 50 SFC per million PBMCs as previously reported^40,41^. By using this methodology, at least 85% of the responding cells are CD8 T cells, but as all studies were conducted with unfractionated PBMCs, responses are described as HIV-specific T cells.

### Genome analysis of protease gene

A fragment corresponding to the HIV protease gene was obtained by PCR or RT-PCR from DNA isolated from PBMCs or from RNA isolated from plasma using commercial assays (Qiagen) and then deep-sequenced in a GS-Junior instrument using 454 sequencing technology (Roche Life Sciences). On average, 200 sequences by sample were analyzed^25^. Phylogenetic trees (Neiborgh-joining) and average pairwise distances (APD) were estimated with Mega v6.2 software (https://www.megasoftware.net/).

### Ethical statement

Appropriate informed consent was obtained from each subject in accordance with the Spanish legislation in compliance with the confidentiality and privacy rules. The procedures of this study were approved by the Institute Ramón y Cajal for Health Research (IRYCIS) Ethics Committee and by the Spanish Agency for Medications and Health Products (AEMPS) and were carried out in compliance with the Helsinki Declaration.
